# Loss of c-KIT expression in thyroid cancer cells

**DOI:** 10.1371/journal.pone.0173913

**Published:** 2017-03-16

**Authors:** Sara Franceschi, Francesca Lessi, Federica Panebianco, Elena Tantillo, Marco La Ferla, Michele Menicagli, Paolo Aretini, Alessandro Apollo, Antonio Giuseppe Naccarato, Ivo Marchetti, Chiara Maria Mazzanti

**Affiliations:** 1 FPS—Pisa Science Foundation, Pisa, Italy; 2 Department of Translational Research and New Technologies in Medicine and Surgery, University Hospital of Pisa, Pisa, Italy; 3 Department of Pathology, University of Pittsburgh School of Medicine, Pittsburgh, PA, United States of America; 4 Core Research Laboratory, Istituto Toscano Tumori, Firenze, Italy; University of South Alabama Mitchell Cancer Institute, UNITED STATES

## Abstract

Papillary thyroid carcinoma is the most frequent histologic type of thyroid tumor. Few studies investigated the role of c-KIT expression in thyroid tumors, suggesting a role for this receptor and its ligand in differentiation and growth control of thyroid epithelium and a receptor loss following malignant transformation. We investigated and correlated c-KIT expression levels and two known markers of thyrocytes differentiation, PAX8 and TTF-1, in malignant and benign cytological thyroid samples. Moreover, we performed functional studies on human papillary thyroid carcinoma cell line to associated c-KIT expression to thyrocytes differentiation and tumor proliferation. c-KIT and PAX8 expression resulted higher in benign samples compared to the malignant ones, and the expression levels of these two genes were significantly correlated to each other. We also observed that c-KIT overexpression led to an increase of PAX8 expression level together with a decrease of proliferation. Furthermore, c-KIT overexpressing cells showed a regression of typical morphological features of malignancy. Taken together these results suggest that c-KIT could be involved in the differentiation of thyroid cells and in tumor progression.

## Introduction

Papillary thyroid carcinomas (PTC), the most common form of thyroid cancers, represents the majority of thyroid carcinomas (~ 80–90%) [1]. Until now, a large amount of information has been collected on the molecular pathogenesis of PTC, in particular, it is well known the importance of the activation of the mitogen-activated protein kinase (MAPK) pathway, due to point mutations of the BRAF and RAS genes and RET/PTC rearrangements [2–4].

The proto-oncogene c-KIT encodes for the tyrosine kinase receptor (CD117) and is involved in cell signal transduction with different downstream pathways: MAPK, phosphatidylinositol 3-kinases (PI3K), Janus kinase (JAK)/signal transducers and activators of transcription (STAT), SRC family kinases (SFK) and phospholipase Cγ [5]. Furthermore, c-KIT is a mutagenic effective proto-oncogene with a stem-cell factor (SCF) as a ligand, and it leads to tumor growth through impairment of cellular growth regulation [6].

However, the precise role of c-KIT in human tumors remains largely unknown and data from the literature present discrepancies depending on the tumor type. There are papers showing that c-KIT is highly expressed or mutated in small cell lung cancer [7], leukemia cells [8], colon cancer [9] and neuroblastoma [10]. On the other hand, c-KIT expression is lost in breast cancer [11] and melanoma [12]. Some studies investigated c-KIT expression in thyroid gland and in thyroid malignancies [13–16], suggesting a role for this receptor and its ligand in differentiation and growth control of thyroid epithelium and that this function may be lost following malignant transformation. In particular, in our previous paper [17] we evaluated c-KIT expression in a group of thyroid fine-needle aspiration cytology (FNAC) smears, showing that c-KIT analysis improves the cytological diagnostic accuracy, and we confirmed the down regulation of c-KIT in PTC comparing to benign nodules (BN). Nowadays, FNAC remains the most reliable, cost-effective, and safe diagnostic method for the evaluation of thyroid nodules and the cytological examination of the obtained material is the best single test for differentiating malignant from benign thyroid nodules reducing the need for thyroid surgery [18–20].

In the present study, we decided to explore the role of c-KIT in thyroid tumor proliferation and differentiation by analyzing two known markers of thyrocytes differentiation: PAX8 (Paired-box gene 8) and TTF-1 (Thyroid transcription factor-1) [21, 22]. Starting from 69 FNAC smears with known c-KIT expression levels, previously described in our paper [17], we here investigated PAX8 and TTF-1 mRNA expression levels. Moreover, we overexpressed c-KIT in a PTC cell line to perform functional studies.

## Materials and methods

### FNAC specimens

69 Preoperative FNAC slides of thyroid nodules, collected from as many patients from 2003 to 2010, were selected from the archives of the Division of Surgical, Molecular and Ultrastructural Pathology, Santa Chiara University Hospital, Pisa.

### Ethical board

The study was approved by the Ethics Committee of University Hospital of Pisa and signed informed consent was obtained from each of the subjects. All methods were performed in accordance with approved guidelines.

### Diagnosis

The histological diagnosis of the 69 samples collected was of BN in 39 cases and PTC in 30 cases. In all cases, FNAC was done under ultrasonographic guidance and the cytological diagnosis was carried out as previously described [17]. Smears were independently reviewed by senior cytopathologists to assure adequate thyroid cell representation of the slides in which molecular analysis was performed.

### RNA extraction from FNAC

Archival FNAC slides stained with Papanicolaou technique were kept in xylene to detach slide coverslips. The slides were then hydrated in a graded series of ethanol followed by a wash in distilled H_2_O and finally air-dried. RNA extraction was performed using the High Pure RNA Paraffin kit (Roche, Basel, Switzerland) scraping off the cytological stained sample with the lysis buffer. The quantity/quality of RNA was estimated with Nanodrop 1000 spectrophotometer (Thermo Fisher Scientific, Waltham, MA, USA) using 1 μl of undiluted RNA solution. RNA was treated with DNase Ι recombinant, RNase-free (Roche, Basel, Switzerland).

### RNA extraction from cells

Total RNA was extracted from K1 cultured cells with the automated system Maxwell 16 (Promega, Madison, WI, USA) after 48 h from transfection following the Maxwell 16 LEV simplyRNA Cells Kit manufacture’s protocol. The quantity and quality of extracted RNA was estimated with Qubit 2.0 Fluorometer (Life Technologies, Foster City CA, USA) by using 2 μl of undiluted RNA solution.

### c-KIT, PAX8 and TTF-1 mRNA expression analysis

RNA was reverse transcribed using the AMV Reverse Transcriptase, cloned kit (Invitrogen, Carlsbad, CA, USA) in a final volume of 20 μl, containing 5X RT buffer, 10 mM dNTPs, 50 ng/μl Random Primers, 0.1 M DTT, 40 U/μl RNaseOUT, 50 μ M oligo(dT), DEPC-Treated Water, 15 U/μl Cloned AMV reverse transcriptase.

The level of c-KIT, PAX8 and TTF-1 mRNA was analyzed by quantitative Real Time PCR (qPCR) on the Rotor Gene 6000 real time rotary analyzer (Corbett Life Science, Sidney, Australia) following the manufacturer’s instructions. Endogenous reference gene (B_2_M, beta 2 microglobulin) was used to normalize each gene expression level. PCR was performed in 25 μl final volume, containing 5 μl of cDNA, 12.5 μl of MESA GREEN qPCR MasterMix Plus (Eurogentec, San Diego, CA, USA), 300 nM of each primer (Invitrogen, Carlsbad, CA, USA) with the following cycling conditions: initial denaturation 95°C for 5 min; 40 cycles at 95°C for 15 sec and 58°C for 40 sec and 72°C for 40 sec; final step 25°C for 1 min. Each samples was performed in triplicate. The following primers were used: c-KIT, 5’-GCACCTGCTGCTGAAATGTATGACATAAT-3’, 5’-TTTGCTAAGTTGGAGTAAATATGATTGG-3’, PAX8, 5’-GCCCAGTGTCAGCTCCATTA-3’, 5’-GAGGTTGAATGGTTGCTGCA-3’, TTF-1, 5’-GATGTCCTCGGAAAGTCAGC-3’, 5’-CTCCAGGGGACTCAAGATGT-3’.

### Cell culture and transfection

Human thyroid carcinoma K1 cell line, not expressing c-KIT, was obtained from Sigma Aldrich (Sigma Aldrich Cat# 92030501, RRID:CVCL_2537) (Sigma Aldrich, Saint Louis, MO, USA). Cells were cultured in DMEM:Ham′s F12:MCDB 105 (2:1:1) (Thermo Fisher Scientific, Waltham, MA, USA) + 2mM Glutamine (Thermo Fisher Scientific, Waltham, MA, USA) + 10% Fetal Bovine Serum (FBS) (Thermo Fisher Scientific, Waltham, MA, USA). The cells were grown under a humidified condition at 37°C in 5% CO_2_.

A plasmid pBluescriptR containing c-KIT gene was obtained by Source BioScience (Source BioScience, Nottingham, UK), then c-KIT gene was isolated and inserted by restriction digestion in a pIRES2-AcGFP vector (Clontech Laboratories, Mountain View, CA, USA) to generate pIRES2-AcGFP-c-KIT. c-KIT sequence was confirmed by Sanger sequencing.

A total of 90,000 K1 cells per well were seeded in a 6-well plate and transfected with pIRES2-AcGFP-c-KIT (c-KIT+ cells), with the empty vector (c-KIT- cells) and with only Lipofectamine (control) using Lipofectamine 3000 (Thermo Fisher Scientific, Waltham, MA, USA) according to manufacturer’s protocol.

### Flow cytometry analysis

Flow cytometry was used to detect cells transfected with p-IRES-AcGFP and p-IRES-AcGFP-c-KIT plasmids using CyFlow® Cube 8 Sorter Flow Cytometer (Sysmex Partec, Gorlitz, Germany) and evaluate transfection efficiency.

2x10^7^ cells per sample were washed with PBS and incubated with PE conjugated monoclonal mouse antibody anti-CD117 (Miltenyi Biotec Cat# 130-091-734, RRID:AB_615058) (Miltenyi Biotec, Calderara di Reno, BO, Italy) following the manufacturer’s instructions.

Each sample was analyzed in the same run, with identical settings equipped with a blue diode pumped solid-state Laser (20mV) at 488 nm. At least 10,000 events per samples were collected and the gating strategy was based on Forward Scatter (FSC) and Side Scatter (SSC) characteristics. Data analysis was performed using FCS express 4 software (De Novo Software™).

### Immunocytochemistry

c-KIT+ and c-KIT- cells suspensions were dropped and spread over the glass-slide. Then the slides were fixed with 3.7% formaldehyde for 10 minutes and washed three times in PBS. Cells were permeabilized with 0.1% Triton X-100 in and washed three times in PBS. Immunocytochemistry was performed using the Mouse specific HRP/DAB (ABC) Detection IHC Kit (Abcam, Cambridge, UK) according to manufacturer’s protocol. The antigen unmasking was achieved with MS-unmasker solution (DIAPATH, Martinengo, BG, Italy) in microwave. Primary antibody, mouse monoclonal c-KIT (Santa Cruz Biotechnology Cat# sc-13508, RRID:AB_626874) (Santa Cruz Biotechnology, Dallas, TX, USA), was used at 1:200 dilution for 1 h at room temperature. Slides were developed with diaminobenzidine chromogen (DAB) (DAKO, Glostrup, DK) and counterstained with hematoxylin. Negative controls included the omission of the primary antibody. Slides were analyzed using the inverted microscope CARL ZEISS Axio Observer Z1FLMot, and images were taken with CARL ZEISS AXIOCAM Icc1 camera.

### WST1 proliferation assay

A total of 7,000 cells per well of each type (c-KIT+, c-KIT- and control cells) were seeded in a 96-well plate format, after 48 h from transfection. After 24 h, 48h and 72 h, the WST1 reagent (Clontech Laboratories, Mountain View, CA, USA) was added and incubated for a further 2 h before reading the plate. Each assay was conducted in sets of eight. The quantity of formazan dye is directly related to the number of metabolically active cells, and was quantified by measuring the absorbance at 450 nm in a multiwell plate reader (Tecan, Mannedorf, Switzerland).

### Morphological analysis

To observe the morphological aspect of c-KIT+ and c-KIT- cells, we spread a drop from each cell suspensions over the glass-slide. Then the slides were allowed to air dry for 30 minutes and fixed for 10 minutes with 4% formaldehyde. At the end the slides were stained with Papanicolaou stain and reviewed by a senior cytopathologist under the inverted microscope CARL ZEISS Axio Observer Z1FLMot, who evaluated the main characteristics of malignancy: nuclear pleomorphism and hyperchromasia, ratio nucleus/cytoplasm, coarse chromatin pattern, multinucleation, pleomorphism of nucleoli and presence of multiple nucleoli. Images were taken with CARL ZEISS AXIOCAM Icc1 camera.

### Statistical analysis

The expression levels of PAX8, TTF-1 in FNAC samples and K1 cells were statistically analysed by unpaired Student’s t-test. Regression analysis was used to assess the relationship between two gene expression levels and to predict their correlation. These analyses were all performed by using MedCalc for Windows, version 12 (MedCalc Software, Mariakerke, Belgium). Data sets derived from WST1 analysis were screened by one-way analysis of variance analysis (ANOVA).

## Results

### PAX8 and TTF-1 expression levels in patients

**Fig 1:**
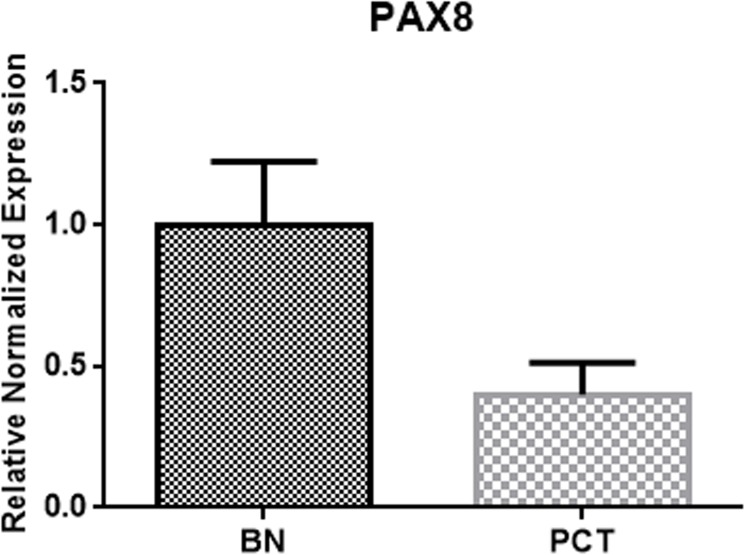
PAX8 overexpression in benign thyroid nodules. PAX8 mRNA expression in PTC (n = 39) and BN (n = 30) (FNAC) samples.

PAX8 expression level was significantly higher in the benign group (BN; n = 30) compared to the malignant group (PTC; n = 39) (p < 0.01) (Fig 1 and S1 Table).

**Fig 2:**
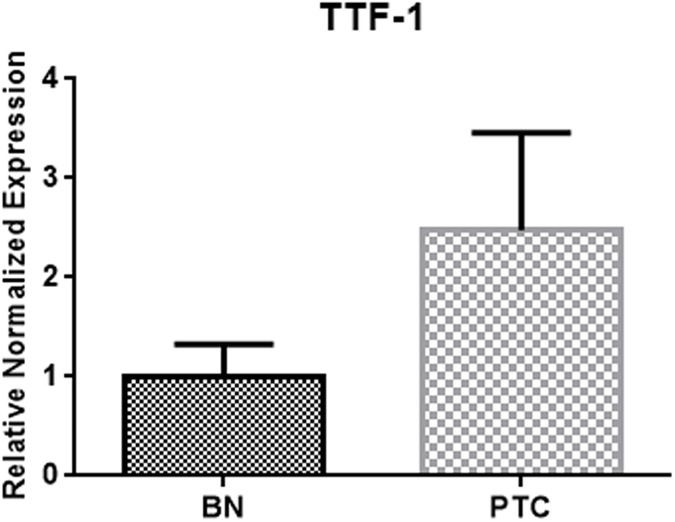
TTF-1 downregulation in benign thyroid nodules. TTF-1 mRNA expression in PTC (n = 39) and BN (n = 30) (FNAC) samples.

The TTF-1 gene had a significantly lower expression in the benign group (BN; n = 30) compared to the malignant group (PTC; n = 39) (p < 0.01) (Fig 2 and S1 Table).

### PAX8 and TTF-1 correlation with c-KIT expression

c-KIT mRNA expression was evaluated on the same case series of our previous paper [17], finding that c-KIT was more expressed in benign patients than in malignant ones.

**Fig 3:**
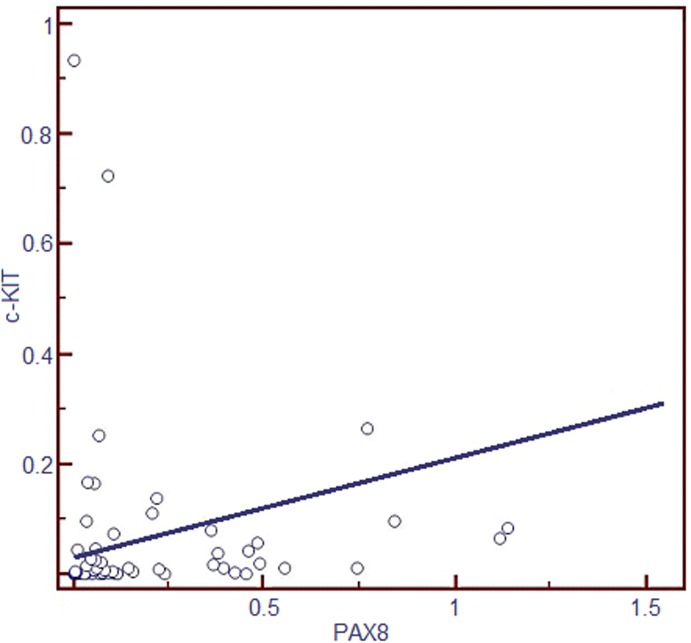
Regression analysis between and gene expression in 69 thyroid nodules including 30 BN and 39 PTC. Data are expressed in relative normalized expression levels after normalization by B_2_M gene expression. High c-KIT mRNA levels were associated with a clear increase of PAX8 mRNA level.

Regression analysis showed a statistically significant (p = 0.003) correlation between c-KIT and PAX8 with a correlation coefficient of r = 0.4 and a coefficient of determination r^2^ = 0.13 (Fig 3). No statistically significant correlation was found between c-KIT and TTF-1 expression levels (data not shown).

### Cellular proliferation and morphological analysis in c-KIT overexpressing cells

**Fig 4:**
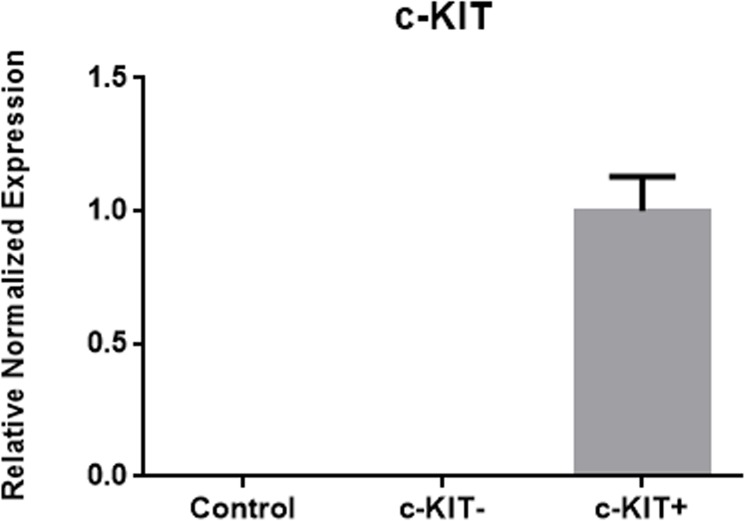
c-KIT overexpression in c-KIT+ transfected cells. c-KIT mRNA expression in control, empty vector transfected cells and c-KIT overexpressing cells.

**Fig 5:**
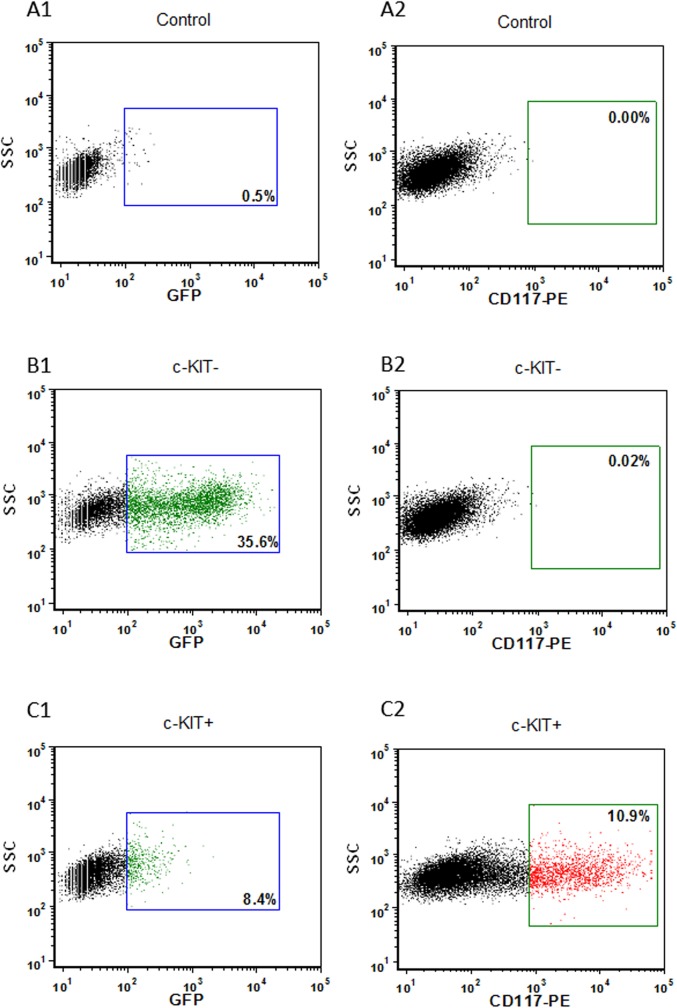
c-KIT overexpression in c-KIT+ transfected cells. Flow cytometric analysis of c-KIT expression in control, empty vector transfected cells and c-KIT overexpressing cells. Data are represented in dot plots of GFP (FL1) vs. SSC and CD117-PE (FL2) vs. SSC. Non-transfected cells (A1-2), empty vector transfected cells (B1-2) and c-KIT overexpressing cells (C1-2). Blue regions in A1, B1 and C1 plots represent the percentage of GFP fluorescent cells (transfection efficiency). Green gates in A2, B2 and C2 plots show red fluorescent cells labeled with PE antibody (c-KIT expression).

**Fig 6:**
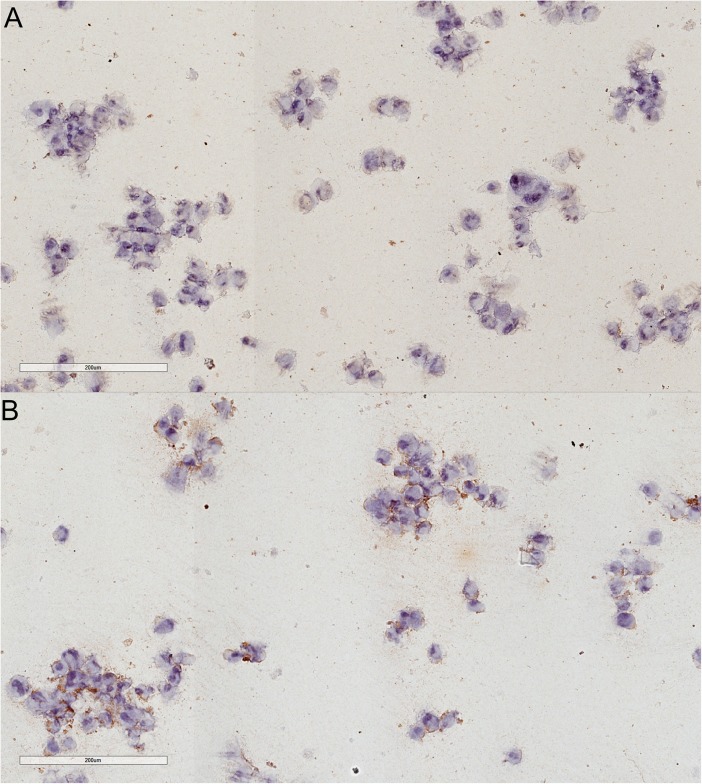
c-KIT overexpression in c-KIT+ transfected cells. Immunocytochemistry staining for c-KIT in in empty vector transfected cells (A) and c-KIT overexpressing cells (B).

c-KIT overexpression in K1 thyroid cancer cells was confirmed by real time PCR, flow cytometric analysis and immunocytochemistry (Figs 4–6 and S1 Table).

**Fig 7:**
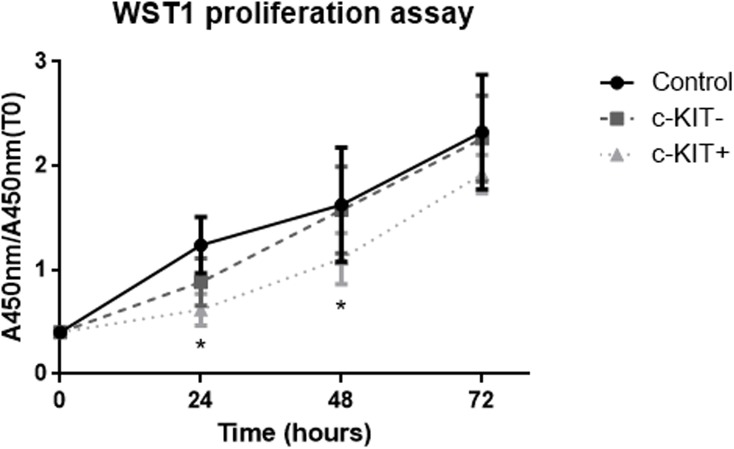
Effect of c-KIT overexpression on cell proliferation. WST-1 based proliferation assays were performed for control, empty vector transfected cells and c-KIT overexpressing cells.

Then, we investigated cell proliferation following c-KIT overexpression for three days by WST-1 assay. The proliferative index of c-KIT+ cells was 1.43-fold lower than c-KIT- cells at 24h (p = 0.0170), 1.42-fold lower at 48h (p = 0.0164) and 1.18 fold lower at 72h (not significant) (Fig 7 and S1 Table).

Cells were seeded in 96-well plate after 48 h from transfection and cell proliferation was measured by WST-1 assay at time 0 and every 24 h thereafter, up to 72 h. Data are expressed as means ± SD; N = 8; *p < 0.05.

**Fig 8:**
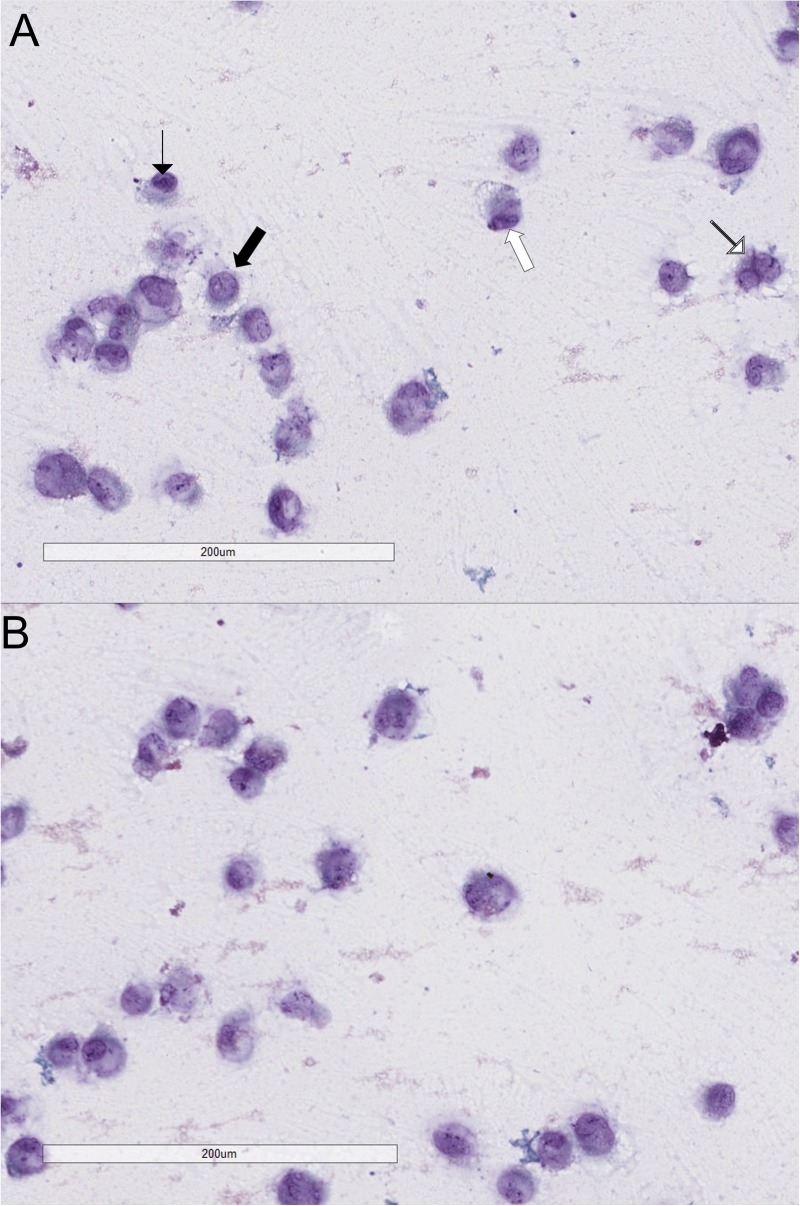
Effect of c-KIT overexpression on cell morphological characteristics. Four principal malignancy features were more represented in empty vector transfected cells (A) than c-KIT overexpressing cells (B): high nuclear-cytoplasmic ratio (black thick arrows); presence of powdery chromatin pattern, sometimes with clumps (black thin arrows) presence of macro and multiple nucleoli (white thick arrows) cells presenting more nuclei (2 or 3).

An assessment of the morphological characteristics was performed by observing c-KIT- and c-KIT+ cell slides under the microscope after the Papanicolaou staining. Comparing c-KIT- and c-KIT+ cells we found four principal cell features, peculiar characteristics typical of malignancy, more represented in c-KIT- cells compared to c-KIT+ cells (Fig 8): high nuclear-cytoplasmic ratio; presence of powdery chromatin pattern, sometimes with clumps; presence of macro and multiple nucleoli; cells presenting more nuclei (2 or 3).

**Fig 9:**
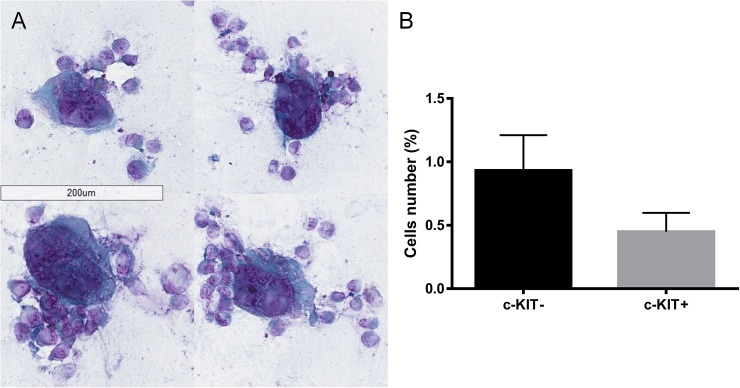
Presence of atypical cells in c-KIT- and c-KIT+ cells. Abnormal cells (A) in empty vector transfected cells and c-KIT overexpressing cells (B).

Moreover, we observed the presence of highly atypical cells (Fig 9A) in both c-KIT+ and c-KIT- cells slides. Those abnormal cells were counted for the entire surface of c-KIT- and c-KIT+ slides (performed in triplicate) by three different operators finding a 2.07-fold greater percentage of atypical cells in c-KIT- cells compared to c-KIT+ cells (p = 0.0003; Fig 9B and S1 Table).

### PAX8 expression levels in c-KIT- and c-KIT+ cells

**Fig 10:**
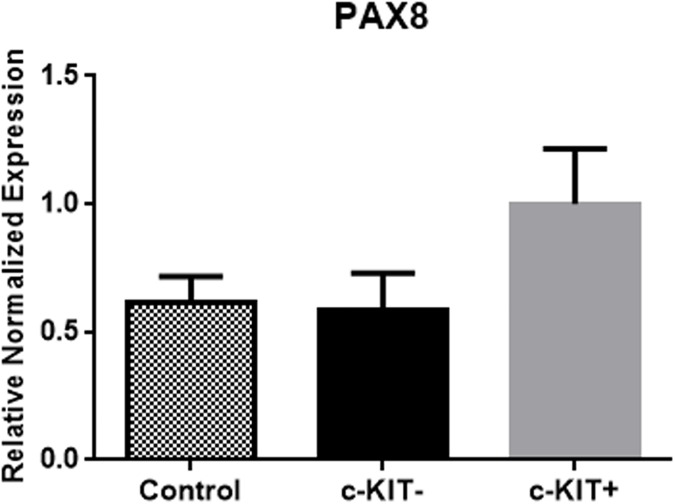
PAX8 overexpression in c-KIT+ transfected cells. PAX8 mRNA expression in empty vector transfected cells and c-KIT overexpressing cells.

PAX8 expression in c-KIT overexpressing cells was 1.8-fold higher than empty vector transfected cells and 1.7-fold higher than control cells (p = 0.0236) (Fig 10 and S1 Table).

## Discussion

This work aims to investigate the role of c-KIT expression in thyroid cancer, especially in PTC, which is still unknown. KIT receptor is involved in the development of hematopoietic stem cells, melanocytes, germ cells, mast cells, and interstitial cells of Cajal [23, 24], but its role in malignant transformation and tumor growth remains largely unexplored. c-KIT mutations can activate the signal transduction cascades that regulate cell proliferation, apoptosis, chemotaxis and adhesion [25]. In GIST tumors, activating mutations of c-KIT were detected, but also the loss or down-regulation of the c-KIT can be observed linked to neoplastic transformation as for example in breast carcinoma and melanoma [11, 12]. Few and dated studies have analyzed c-KIT expression profiles in thyroid tumors [13–16], finding a correlation with differentiation and growth control of thyroid epithelium and also suggesting a c-KIT loss of function in malignant transformation.

In 2004 Mazzanti et al. [26] by microarray assay detected c-KIT out of thousands of genes as one of the most significantly down-regulated genes in PTC compared to BN, moreover, in 2012 our previous paper [17] confirmed the down-expression of c-KIT mRNA in PTC highlighting its importance as a diagnostic marker in thyroid FNAC. More recently, other studies have been published showing a decreased c-KIT expression in PTC compared to normal thyroid tissues [27, 28]. Several mechanisms have been described that can mediate the downregulation of c-KIT, including dysregulated expression of specific microRNAs [29] (miR-146b, miR-221, and miR-222) and promoter hypermethylation [12]; however, there are no studies that investigate this events in thyroid tumors.

Based on these previous studies, we decided to investigate the role of c-KIT expression in PCT carcinogenesis and thyroid cell differentiation. Two known markers of thyrocytes differentiation [21, 22], PAX8 and TTF-1, were analyzed in PCT and BN and correlated with c-KIT expression. PAX8 mRNA expression resulted to be significantly overexpressed in benign samples compared to the malignant group and its expression correlated with c-KIT, with a coefficient of determination that indicates that c-KIT expression is responsible for 13% of PAX8 expression variability. The functions of the nuclear protein PAX8 are linked to the development of the thyroid follicular cells and the expression of thyroid-specific genes [30] and this result promotes the hypothesis that c-KIT may be involved in thyrocytes differentiation. Not significant correlation was found between c-KIT and TTF-1 expression levels, even though we observed a statistically significant downregulation of TTF-1 in benign samples compared to the malignant group.

To the best of our knowledge, this is the first work that investigates the role of c-KIT by overexpressing it in a PTC cell line. Even if the efficiency of transfection was around 10% (evaluated through flow cytometric analysis), c-KIT mRNA and protein expressions levels in c-KIT+ cells were clearly higher than c-KIT- cells. Through the WST1 analysis, we showed that c-KIT overexpression leads to a significant inhibition of cellular proliferation. Moreover, c-KIT- cells showed a greater number of cells with particular morphological characteristics, typical of malignancy, such as high nuclear-cytoplasmic ratio, presence of powdery chromatin pattern, macro and multiple nucleoli, multinucleated cells and presence of abnormal cells than c-KIT+ cells. Furthermore, c-KIT+ cells, demonstrated a significantly higher PAX8 expression level comparing to c-KIT- cells. Taken together these results suggest that c-KIT overexpression may led to thyroid cancer cells regression of malignant features and tumor proliferation. In addition, the positive significant correlation between c-KIT and the thyrocyte differentiation marker PAX8 mRNA levels highlights even more c-KIT possible involvement in cellular differentiation.

Additional studies are needed, however, to further explore the implications of these findings; in particular, to determine the possible mechanisms inducing c-KIT downregulation and to identify other components of the cascade molecular pathway controlled by KIT receptor in order to better understand thyroid cancer development. Furthermore, these findings strengthen the importance of c-KIT expression as a marker of thyroid malignancy. Fine-needle aspiration cytology (FNAC) is an easy, cost-effective test for cancer diagnosis, and its use has strikingly reduced the number of unnecessary thyroidectomy [31]. Unfortunately, about 30% of FNAC results indeterminate and thyroid surgery is required to establish the diagnosis [32]. Therefore, because of this obvious limitation of FNA cytology in the preoperative diagnosis, there is a clinical need for reliable preoperative molecular markers to distinguish benign from malignant thyroid nodules. In this line, this study confirms the diagnostic potential of c-KIT expression as an adjunctive marker in the preoperative management of thyroid nodules.

## Supporting information

S1 Table Individual data points. (XLSX)
